# Crystal structure of *Porphyromonas gingivalis* peptidylarginine deiminase: implications for autoimmunity in rheumatoid arthritis

**DOI:** 10.1136/annrheumdis-2015-207656

**Published:** 2015-07-24

**Authors:** Anna B Montgomery, Jolanta Kopec, Leela Shrestha, Marie-Laetitia Thezenas, Nicola A Burgess-Brown, Roman Fischer, Wyatt W Yue, Patrick J Venables

**Affiliations:** 1 Kennedy institute of Rheumatology, Nuffield Department of Orthopaedics, Rheumatology and Musculoskeletal Sciences, University of Oxford, Oxford, UK; 2 Structural Genomics Consortium, Nuffield Department of Clinical Medicine, University of Oxford, Oxford, UK; 3 Target Discovery Institute, Nuffield Department of Medicine, University of Oxford, Oxford, UK

**Keywords:** Rheumatoid Arthritis, Ant-CCP, Autoimmunity

## Abstract

**Background:**

Periodontitis (PD) is a known risk factor for rheumatoid arthritis (RA) and there is increasing evidence that the link between the two diseases is due to citrullination by the unique bacterial peptidylarginine deiminase (PAD) enzyme expressed by periodontal pathogen *Pophyromonas gingivalis* (PPAD). However, the precise mechanism by which PPAD could generate potentially immunogenic peptides has remained controversial due to lack of information about the structural and catalytic mechanisms of the enzyme.

**Objectives:**

By solving the 3D structure of PPAD we aim to characterise activity and elucidate potential mechanisms involved in breach of tolerance to citrullinated proteins in RA.

**Methods:**

PPAD and a catalytically inactive mutant PPAD^C351A^ were crystallised and their 3D structures solved. Key residues identified from 3D structures were examined by mutations. Fibrinogen and α-enolase were incubated with PPAD and *P. gingivalis* arginine gingipain (RgpB) and citrullinated peptides formed were sequenced and quantified by mass spectrometry.

**Results:**

Here, we solve the crystal structure of a truncated, highly active form of PPAD. We confirm catalysis is mediated by the following residues: Asp130, His236, Asp238, Asn297 and Cys351 and show Arg152 and Arg154 may determine the substrate specificity of PPAD for C-terminal arginines. We demonstrate the formation of 37 C-terminally citrullinated peptides from fibrinogen and 11 from α-enolase following incubation with tPPAD and RgpB.

**Conclusions:**

PPAD displays an unequivocal specificity for C-terminal arginine residues and readily citrullinates peptides from key RA autoantigens. The formation of these novel citrullinated peptides may be involved in breach of tolerance to citrullinated proteins in RA.

## Introduction

Rheumatoid arthritis (RA) is a chronic inflammatory disease characterised by inflammation and destruction of the joints. The current hypothesis of disease development involves a combination of environmental risk factors in genetically predisposed individuals. Periodontitis (PD) is one such environmental risk factor. The prevalence of PD is increased approximately twofold in RA,^1–3^ and severity correlates with measures of disease severity in RA and anticitrullinated protein/peptide antibodies (ACPA).^4^ In addition, the demonstration of autoantibodies to RA-associated autoantigens in patients with PD^5^ supports the hypothesis that PD may drive the autoimmunity that antedates the onset of RA. However, epidemiological studies that prove this temporal relationship are still awaited.

The discovery of ACPA in RA and the involvement of citrullination in a number of other diseases have resulted in a rapidly expanding field of research into the structure and function of peptidylarginine deiminase (PAD) enzymes, which mediate citrullination by conversion of arginine to citrulline. Of the five human PAD isoforms (PAD1–4 and PAD6) PAD2 and PAD4 are of particular interest as they are often found at sites of pathology, including the RA joint^6^ and PD tissues.^7^ The crystal structures of PAD2 and PAD4 have been solved, guiding the design of inhibitors for use in the treatment of inflammatory and malignant disease.^8^ ^9^ However the essential functions of the eukaryotic PADs in normal physiology mean these agents must be used with great caution, and have high potential for toxicity. The constitutive expression of citrullinated peptides also indicates native citrullination is not sufficient to induce an anticitrulline response.^10^ To this end, citrullination by the only known prokaryotic PAD expressed by keystone periodontal pathogen *Porphyromonas gingivalis* (PPAD) has been implicated in the aetiology of RA.^11^ ^12^ Antibodies to *P. gingivalis* are found in individuals at high-risk of developing RA, and in established disease where an increased anti-PPAD response is also observed.^3^ ^12^ ^13^ PPAD differs from human PADs in a number of ways, including (1) sequence homology limited to key residues in the active site, conserved between members of the guanidino modifying enzyme (GME) superfamily,^14^ (2) a lack of requirement for Ca^2+^ ions during catalysis,^15^ (3) an ability to citrullinate free L-arginine residues^16^ and, notably, (4) a preference for substrates with a C-terminal arginine, provided by an additional *P. gingivalis* enzyme, the arginine specific protease arginine gingipain (Rgp).^17^ PPAD is expressed with Rgp on the bacterial outer membrane, and the requirement for Rgp has been demonstrated by an almost complete lack of citrullination when autoantigens are incubated with Rgp knockout strains of *P. gingivalis.*^18^ These findings indicate the two enzymes act together to generate C-terminally citrullinated peptides.^15^ ^18^ Several studies have shown that PPAD can autocitrullinate^15^ ^18^ ^19^ in a similar way to that of PAD4,^20^ and so may be capable of citrullinating internal arginine residues. However a recent study^19^ has suggested that autocitrullination of PPAD may be an artefact of cloning recombinant enzymes, as PPAD purified from supernatants of *P. gingivalis* cultures did not autocitrullinate.

As a potential candidate for breaking tolerance in RA,^19^ ^21^ PPAD has been well studied but there remains some uncertainty about the details of its catalytic mechanisms. In this study we present the crystal structures of wild type PPAD and a catalytically inactive mutant at 1.46 Å and 1.48 Å resolution, respectively. We applied this structural information to characterise the specificity of PPAD for C-terminal arginine residues and demonstrate potential for PPAD to form novel citrullinated epitopes from RA autoantigens.

## Materials and methods

### Recombinant PPAD production and crystallisation

tPPAD^WT^ amino acid sequence 49-484 was amplified from the full-length PPAD coding sequence of *P. gingivalis* strain W83 using forward (5′AATCCCCCTGCAGGTCCTG) and reverse (5′GGCGTTGAACCATGCACGAA) primers with upstream (5′TACTTCCAATCCATG) and downstream (5′TATCCACCTTTACTGTCA) 5′ extensions to enable ligation independent cloning into pNIC28-Bsa4 vector (inhouse at Structural Genomics Consortium (SGC), Oxford. GenBank Accession No. EF198106) generating an N-terminal His6-tagged fusion protein. Recombinant proteins were expressed in *Escherichia coli* BL21(DE3)-R3-pRARE2 cells (inhouse SGC) and purified from protein soluble fraction using Talon (Clontech) Co^2+^-nitrilotriacetic acid (NTA) affinity chromatography before further purification by size exclusion chromatography on an S200 column on the ÄKTAxpress system. His-fusion tags were removed by *Tobacco Etch Virus* cleavage (inhouse SGC) using 1 mg/20 mg protein and incubated overnight (o/n) before Co^2+^-NTA affinity chromatography was repeated to remove free fusion tag.

Site-directed mutagenesis using the megaprimer method was carried out as described^22^ to create a number of mutants. To generate tPPAD^C351A^, Cys351 was replaced with Ala and to generate tPPAD^R152A^ and tPPAD^R154A^, Arg152 and Arg154 were replaced with Ala. Further cloning and purification strategies for tPPAD^C351A^, tPPAD^R152A^ and tPPAD^R154A^ remained the same as tPPAD^WT^. Constructs were sequenced to confirm insertion of DNA segments and that mutations were successful.

PPAD was crystallised by vapour diffusion at 20°C. Diffraction data were collected at the Diamond Light Source. Structures of PPAD were solved by molecular replacement.

Recombinant human PAD2 and PAD4 were cloned, expressed and purified as previously described.^9^ ^23^

Protein concentrations of all PPAD and PAD enzymes were standardised using Bradford assay and NanoDrop A280 module.

### Quantification of enzyme activity

The colorimetric assay for citrullination activity was used as previously described,^24^ with synthetic arginine substrate benzoyl arginine ethyl-ester (BAEE) (Sigma), synthetic peptides Arg-Gly-Glu, Met-Arg-Phe, Gly-Arg or fibrinogen peptides FibA-R (CESSSHHPGIAEFPS-R) and FibA-R-XX (CESSSHHPGIAEFPS-R-GK) (Pepceuticals).

### Sample preparation and mass spectrometric analysis

Following in vitro citrullination, proteins were precipitated using chloroform/methanol.^25^ The aqueous supernatant was dried in a vacuum concentrator followed by desalting and analysis as described previously.^10^ Citrullinated peptides were manually inspected for correct precursor mass and fragment annotation to exclude false positives.

More detailed materials and methods can be found in online supplementary information.

## Results

### Recombinant production of PPAD wild type and catalytic mutant

**Figure 1:**
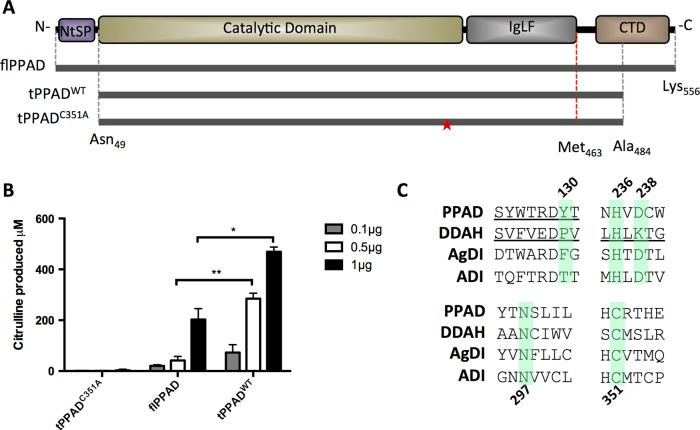
Characterization of tPPAD^WT^ and tPPAD^C351A^ compared with flPPAD. (A) Full length PPAD (flPPAD) (Met1-Lys556), cloned (grey dotted line; Asn49-Ala484) and structurally visible (red dotted line, Asn49-Met463) regions of tPPAD^WT^ and tPPAD^C351A^. Mutation of Cys351 in tPPAD^C351A^ is indicated by ★. (B) Activity of tPPAD^WT^ and tPPAD^C351A^ compared with flPPAD measured by citrulline production from benzoyl arginine ethyl-ester (BAEE). Values are mean N=2±SD *p≤0.05, **p≤0.005. (C) Sequence conservation within guanidino modifying enzyme superfamily. Highlighted residues are conserved and known/predicted to be involved in catalysis. PPAD, *P. gingivalis* peptidylarginine deiminase; DDAH, dimethylarginine dimethylaminohydrolase; AgDI, agmatine deiminase; ADI, arginine deiminase.

PPAD is organised into four domains: the N-terminal signal peptide (NtSP, aa 1-48), catalytic/deiminase domain (CD, aa 49-360), Ig-like fold (IgLF, aa 361-463) and C-terminal domain (CTD, aa 464-556)(figure 1A). A library of PPAD constructs with different fusion tags and N-terminal /C-terminal truncations was tested for recombinant expression in *E. coli*. Among them, a His_6_-tagged truncated construct (Asn49-Ala484, tPPAD^WT^), encoding CD and IgLF but lacking NtSP and CTD, yielded highly soluble and crystallisable protein, with significantly increased catalytic activity towards the in vitro arginine substrate *N*_α_-Benzoyl-L-arginine ethyl ester hydrochloride (BAEE) as compared with full length PPAD (figure 1B). An equivalent mutant construct (tPPAD^C351A^) with the essential catalytic nucleophile among GMEs Cys351^26^ (figure 1C) substituted to Ala, is catalytically inactive (figure 1B). Attempts to express constructs containing CD only were unsuccessful.

### The PPAD CD and IgLF domains form a tightly packed structure

**Figure 2:**
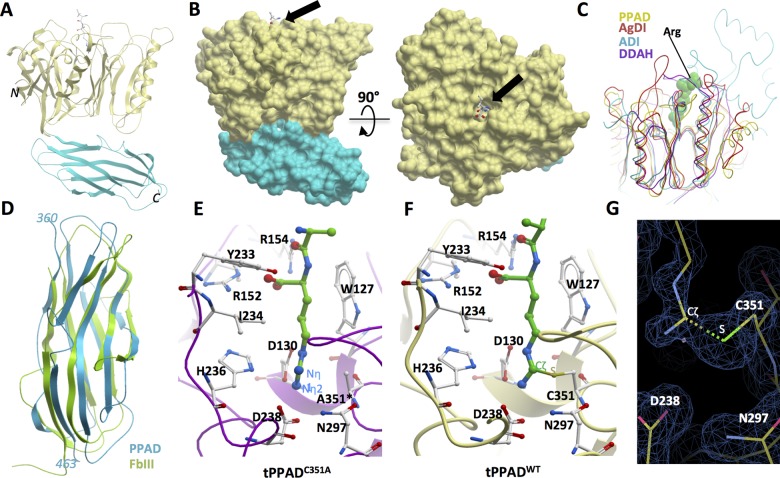
Structures of tPPAD^WT^ and tPPAD^C351A^. (A) The crystallised structure comprises two domains; deiminase domain aa490-360 (yellow) and Ig-like fold aa361-461 (blue). See online supplementary information figure S1 for secondary structure assignment. (B) Surface representation of PPAD showing the arginine-binding pocket in orthogonal views. (C) Structural superposition of guanidino modifying enzyme (GME) members. PPAD, *P.gingivalis* peptidylarginine deiminase; DDAH, dimethylarginine dimethylaminohydrolase; AgDI, agmatine deiminase; ADI, arginine deiminase. The arginine ligand from tPPAD^C351A^ structure is shown to indicate the active site. (D) Structural superposition of PPAD IgLF with fibronectin type III (FbIII). E) The active site of tPPAD^C351A^ with a bound C-terminal arginine. Key residues for substrate binding and catalysis are shown in sticks. (F) The active site of tPPAD^WT^ with a covalently bound amidino adduct. (G) Amidino adduct of tPPAD^WT^ with its Cζ atom covalently linked to the Cys351 S atom, as revealed by 2Fo-Fc electron density (blue mesh lines).

We have determined the 1.46 Å and 1.48 Å structures of tPPAD^WT^ and tPPAD^C351A^ by molecular replacement (see online supplementary information table S1), which reveal identical and superimposable conformations (root mean square deviation (RMSD)∼0.09 Å), suggesting that the Cys351Ala substitution did not perturb protein integrity. The visible PPAD structure comprises aa 49-463 arranged into the CD and IgLF regions (figure 2A; see online supplementary information figure S1). The CD adopts the canonical fold of ββαβ motifs conserved among GME structures (figure 2C)(RMSD 2.0–2.4 Å). The active site entrance is located on one face of the CD (figure 2B) and harbours shorter, less extensive connecting loops as compared with other GMEs. The opposite face of the CD mediates extensive interdomain contacts with the IgLF, a β-sandwich motif absent in the other GMEs, bearing limited structural homology to a variety of fibronectin type III proteins (RMSD 2.0–2.8 Å)(figure 2D)

### Snapshots of catalysis with active site bound ligands

The PPAD active site is an elongated cavity with its entrance exposed to the surface exterior (figure 2B). Difference Fourier electron density maps clearly reveal an active site-bound ligand, modelled in the tPPAD^C351A^ structure as an arginine-containing dipeptide (figure 2E), and in the tPPAD^WT^ structure as an amidino adduct where its Cζ atom is covalently linked to the Cys351 S atom (figure 2F,G). Both ligands fit snugly within the active site cavity, using their hydrocarbon moiety to pack against the hydrophobic cavity wall (lined by Trp127, Ile234), and their terminal carboxylate oxygens to form ionic interactions with Arg152 and Arg154 as well as hydrogen bond with Tyr233 at the active site entrance. At the end of the cavity, the site of the PPAD citrullination reaction, both ligands are coordinated by five polar residues strictly conserved among GMEs (figure 1C), namely Asp130, His236, Asp238, Asn297 and Cys351/Ala351. The acidic residues Asp238 and Asp130 serve to fixate the arginine guanidino Nη1 and Nη2 atoms by ionic interactions (figure 2E), thereby positioning the Cζ atom for a nucleophilic attack by the Cys351 sulphuryl group (as evidenced from the Cζ-S thioether bond in the tPPAD^WT^ structure, figure 2G). The imidazole group of His236 is in proximity to and at the opposite face of the guanidino plane from Cys351, and could act as a general acid/base for proton transfer (see figure 2F and online supplementary information figure S2).

### PPAD has distinct substrate specificity from human PAD2 and PAD4

**Figure 3:**
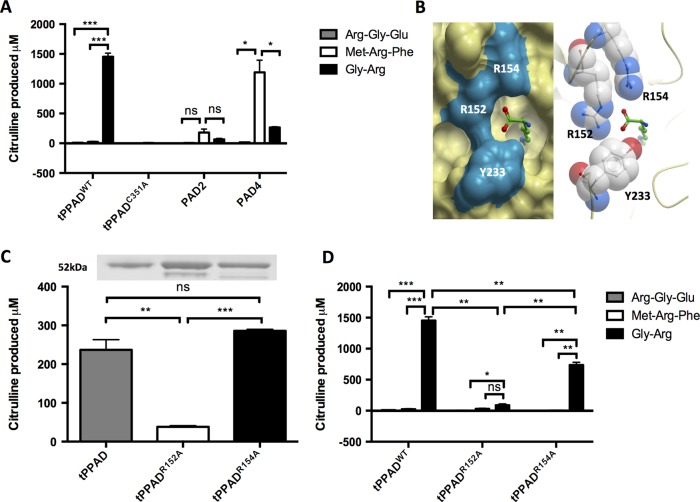
Substrate specificity of tPPAD, PAD2 and PAD4. (A) 1 μg tPPAD^WT^, tPPAD^C351A^, PAD2 or PAD4 was incubated with 5 mM synthetic peptide for 1 h at 37°C and citrulline produced measured. (B) Structural view of the PPAD active site entrance showing role of R152, R154 and Y233 in binding terminal carboxyl group of arginine. (C) 3 μg tPPAD^WT^, tPPAD^R152A^ or tPPAD^R154A^ was incubated with 20 mM benzoyl arginine ethyl-ester (BAEE) for 1 h at 37°C and citrulline produced measured. Standard expression, purity and concentration of protein were confirmed by sodium dodecyl sulphate-polyscrylamide gel electrophoresis (SDS-PAGE) and Coomassie staining (upper panel). (D) 1 μg tPPAD^WT^, tPPAD^R152A^ and tPPAD^R154A^ were incubated with 5 mM synthetic peptide for 1 h at 37°C and citrulline produced measured. For all graphs values are mean n=2±SD *p≤0.05, **p≤0.005, ***p≤0.005.

To examine the substrate specificities of PPAD compared with human PAD2 and PAD4, colorimetric detection of citrulline was used with three short peptides containing either N-terminal (Arg-Gly-Glu), internal (Met-Arg-Phe) or C-terminal (Gly-Arg) arginine residues following incubation with tPPAD^WT^, tPPAD^C351A^, PAD2 or PAD4 (figure 3A). As expected, tPPAD^WT^ only produced citrulline from Gly-Arg. PAD4 showed significantly higher activity on Met-Arg-Phe than Gly-Arg (p=0.002) and Arg-Gly-Gly (p=0.015). PAD2 displayed a similar trend to PAD4 although did not reach significance due to significantly lower activity overall. tPPAD^C351A^ did not display activity on any of the substrates.

### Mutation of Arg152 and Arg154 inhibits enzyme activity

From our structural data, the arginine terminal carboxylate group is fixated by ionic interactions with Arg152 and Arg154 (figure 3B), suggesting a role for these two basic residues in determining the reactivity of PPAD towards a C-terminal arginyl peptide substrate. To investigate the importance of these interactions in PPAD enzyme activity, we mutated Arg152 or Arg154 to Ala, and expressed the resulting mutant proteins (tPPAD^R152A^ and tPPAD^R154A^) as per tPPAD^WT^. Using BAEE as substrate in the colorimetric assay, the activity of tPPAD^R152A^ is significantly reduced compared with tPPAD^R154A^ (p<0.001) and tPPAD^WT^ (p=0.004). There was no significant difference between tPPAD^R154A^ and tPPAD^WT^ (figure 3C). However, when Arg-Gly-Glu, Met-Arg-Phe and Gly-Arg were used in the assay as before, the activity of tPPAD^WT^ became significantly higher than tPPAD^R152A^ and tPPAD^R154A^ (p=0.001 and p=0.005, respectively). All enzymes retained activity on C-terminal arginine peptide Gly-Arg only (figure 3D).

**Figure 4:**
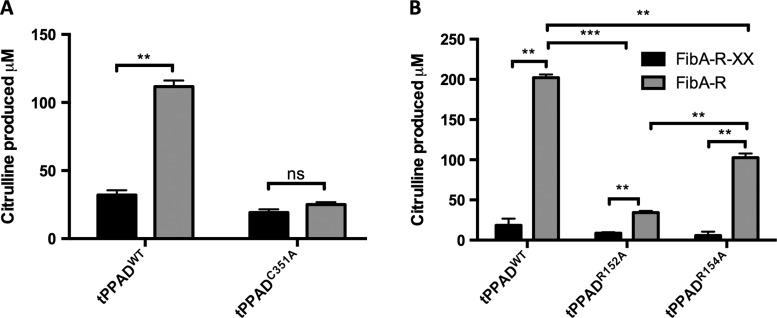
Substrate specificity of wild type and mutant tPPAD on fibrinogen peptides. (A) 1 μg tPPAD^WT^ and tPPAD^C351A^ were incubated with 5 mM fibrinogen peptides at 37°C for 1 h and citrulline production was measured. (B) 1 μg tPPAD^WT^, tPPAD^R152A^ or tPPAD^R154A^ was incubated with 5 mM fibrinogen peptides at 37°C for 1 h and citrulline production measured. In all assays, values are mean n=2±SD *p≤0.05, **p≤0.005, ***p≤0.005.

To examine this result in a more physiologically relevant context, two peptides from the fibrinogen α-chain with a C-terminal (FibA-R: CESSSHHPGIAEFPS-R), or an internal arginine residue (FibA-R-XX: CESSSHHPGIAEFPS-R-GK) were used in the assay. First, level of activity of tPPAD^WT^ and tPPAD^C351A^ on these peptides was compared. Citrullination activity of tPPAD^WT^ on FibA-R was significantly higher than FibA-R-XX (p=0.003), the latter equivalent to the baseline measurement level for inactive tPPAD^C351A^ (figure 4A). Using tPPAD^R152A^ and tPPAD^R154A^ the same patterns of reactivity as with the short synthetic peptides were observed, with activity on FibA-R significantly higher than FibA-R-XX in all groups (figure 4B). As before, activity of tPPAD^R152A^ was significantly lower than tPPAD^WT^ (p=0.0004) and tPPAD^R154A^ (p=0.003). tPPAD^R154A^ was also significantly less active than tPPAD^WT^ on FibA-R (p=0.002). In addition, activity of tPPAD^WT^, tPPAD^R152A^ and tPPAD^R154A^ was lower on longer fibrinogen peptides than short synthetic peptides, with 86%, 39% and 86% less citrulline produced, respectively.

### RgpB and tPPAD^WT^ generate novel C-terminally citrullinated peptides from RA autoantigens

**Figure 5:**
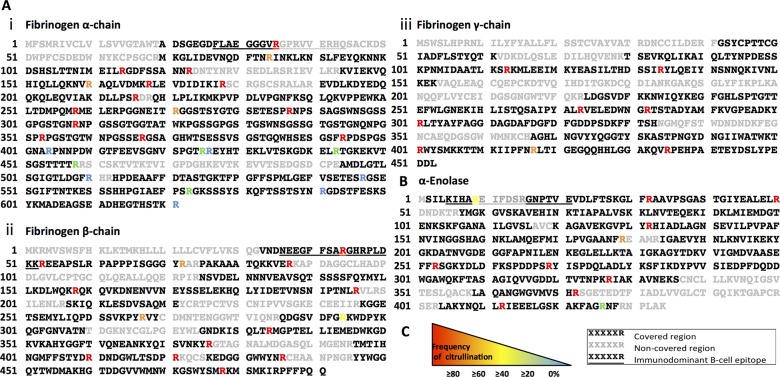
Mass spectrometry analysis of peptides formed by RgpB and PPAD from fibrinogen and α-enolase. Substrates were incubated o/n at 37°C with 10:1 concentration of RgpB and tPPAD^WT^ in ddH_2_O. Peptides formed were identified by liquid chromatography-mass spectrometry (LC-MS), and occurrence of citrullinated variants quantified. (A) Peptides formed from fibrinogen α-chain, β-chain and γ-chain (panels i, ii, and iii, respectively). (B) Peptides formed from α-enolase. (C) Key: Sequence in black denotes region covered in LC-MS result, grey depicts non-covered regions. Underlined sequences denote known immunodominant B cell epitopes. Arginine residues occurring frequently citrullinated are represented by red/orange R, occasionally citrullinated by yellow/green R, and those never detected citrullinated by blue R.

In fibrinogen and α-enolase samples, RgpB was effective in cleaving after every arginine residue of the substrate. tPPAD^WT^ was also highly efficient, citrullinating 21 of the 25 peptides formed from the fibrinogen α-chain (figure 5A), although some occurred in citrullinated form more frequently than others (represented by colour-coded ‘R’ residues in figure 5A, B). All peptides from the fibrinogen β- and γ-chains were citrullinated (14 and 8 respectively, figure 5A(ii),(iii)), and all 11 from α–enolase (figure 5B). In addition, a number of peptides with C-terminal citrullines were identified from tPPAD^WT^ and RgpB (table 1).

**Table 1:** Peptides from tPPAD^WT^ and RgpB identified with C-terminal citrullines

tPPAD^WT^	
R152	DYTGWFAMYDTNKVGLVDFIYNR
R252	YLAPNKILIR
R286	AWGTKYEVYR
R304	ALATNEQPYTNSLILNNR
R445	TYSFTGLNKNDKVEYYISAADNSGR
RgpB	
R263	MIVIVPKKYEEDIEDFVDWKNQR
R518	AQKDGKPTGTVAIIASTINQSWASPMR
R576	KDGEKMLDTWTVFGDPSLLVR

## Discussion

In this study we present the high-resolution crystal structures for active PPAD and a catalytically inactive mutant, revealing the molecular basis of its enzymatic mechanism.

Our structural data reveal the serendipitous trapping of arginine-containing ligands in the PPAD active site. These ligand-bound structures provide snapshots of PPAD activity via the mechanism of Cys nucleophilic hydrolysis proposed for GMEs^27^ (see online supplementary information figure S2). The tPPAD^C351A^ structure captures an intact arginine side chain without enzymatic turnover, mimicking the enzyme-substrate Michaelis complex prior to catalysis. The tPPAD^WT^ structure, revealing an active site covalent adduct, captures a midway step of enzymatic turnover, likely after the Cys351 nucleophilic attack and NH_3_ formation steps. Together, the two structures have confirmed and provided visualisation of the conserved residues known to be involved in catalysis—Asp130, His236, Asp238, Asn297 and Cys351^26^—at the guanidino end of the arginine ligand. We have also discovered additional residues which strongly influence substrate binding and, by extension, enzyme activity: Arg152 and to a lesser extent Arg154. Located away from the site of nucleophilic attack, Arg152 and Arg154 form ionic bonds with the free carboxyl group of a C-terminal arginine, stabilising the substrate for enzymatic turnover. We propose that peptides with an internal arginine would not be favourable substrates for PPAD, as these arginine residues would lack the free carboxyl group necessary for ionic interactions. In addition, residues C-terminal to the arginine residue will sterically clash with the enzyme active site entrance (eg, with Tyr223, figure 3B).

The crystallised tPPAD^WT^ and tPPAD^C351A^ constructs include residues aa464–484, with no visible electron density. This region represents a flexible linker connecting the IgLF and CTD in the full-length polypeptide, and its flexibility likely caused disorder in the crystal. PPAD and RgpB are members of the type-IX secretion system (or PorSS) family, characterised by the dependence on CTD processing for enzyme translocation.^28^ PPAD and RgpB are secreted from *P. gingivalis* in vesicles^29^ in truncated form.^15^ ^30^ In RgpB the CTD requires heavy glycosylation before cleavage,^30^ thus the lack of this machinery during expression of PPAD in *E. coli* may have caused insolubility in the recombinant expression of CD-alone constructs. The truncated form of PPAD secreted from *P. gingivalis* lacks the N-terminal 43 amino acids and CTD, and so has similar N-terminal/C-terminal boundaries to our tPPAD^WT^ construct. If the secreted form is the virulent form of PPAD, the truncation may also increase activity, explaining the increased enzyme activity level compared with full-length PPAD, as we observed.

Using synthetic peptides, we showed that PPAD only citrullinates C-terminal arginine residues, whereas PAD2 and PAD4 both preferentially citrullinated internal arginine residues. In our assays using short synthetic peptides Arg-Gly-GLu, Met-Arg-Phe and Gly-Arg activity of PAD2 was significantly lower than that of PAD4. We suggest this may be due to a preference of PAD2 for longer peptides, which has been observed in our lab (data not shown). We extended this investigation to demonstrate the generation of novel C-terminally citrullinated peptides from key RA autoantigens fibrinogen and α-enolase in combination with RgpB. In a previous study from our laboratory four C-terminally citrullinated peptides from fibrinogen and one from α-enolase were generated by incubating purified proteins with *P. gingivalis* cultures.^18^ No C-terminally citrullinated peptides were obtained when these proteins were incubated with *P. gingivalis* PPAD and Rgp knockout strains, implying a necessity for both enzymes. The current study confirms this directly and increased the number of C-terminally citrullinated peptides observed, to 37 from fibrinogen and 11 from α-enolase. It has also been shown that many of the citrullinated arginine residues identified in this study from fibrinogen and α-enolase can be citrullinated by PAD2 and PAD4 in vitro when in internal positions in the peptide.^31^ ^32^

The strong association of the human leukocyte antigen DRB1 (HLA-DRB1) shared epitope (SE) alleles with an ACPA^+^ phenotype indicates a key role for T cells in RA. Previous studies have examined the preferential binding of citrullinated peptides to major histocompatibility (MHC) shared epitope alleles, leading to T cell activation.^33–35^ In these studies peptides known to react with ACPA, with internal citrulline residues (as would be generated by PAD2 and PAD4) were used. However T cell and B cell epitopes may differ, especially in autoimmune disease. As we have previously proposed^36^ the C-terminally citrullinated peptides generated by PPAD could be recognised as ‘novel’ by T cells, because they are not generated by endogenous PADs. This study has demonstrated formation of many such peptides, and thus strengthens our hypothesis. Further studies are required to investigate if these peptides do stimulate T cells, which is a priority for future work in our laboratory.

Perhaps the most compelling evidence to support the T cell antigenicity of citrullines located at or near the C-terminus is the murine model of autoimmunity in mice transgenic for hen egg lysozyme.^37^ In this model, the immunodominant T cell epitope from _52_DYGILQINSRW_62_, with a C-subterminal citrulline at position 61 is bound to the P10 pocket of the mouse MHC. Interestingly, the resulting antibodies in these mice bound to separate epitopes on native (uncitrullinated) hen egg lysozyme protein. This mechanism could explain why, although patients with PD have a significantly elevated frequency of ACPA, the reaction is not citrulline specific.^5^ ^38^ Similarly, in a separate study antibodies to non-citrullinated peptides occurred before antibodies to the citrullinated variants in patients with presymptomatic autoimmunity destined to get RA.^39^ Together all of these studies suggest citrullination is important in breaking tolerance at the T cell level, while the B cell response may develop from non-citrulline specific to citrulline specific ACPA, as presymptomatic autoimmunity evolves to pathogenic RA.^40^

By solving the crystal structure of the unique pathogen enzyme PPAD, we have elucidated the mechanisms that may cause tolerance breakdown in patients with PD who go on to develop RA. We propose the bacterial origins of PPAD and lack of homology to human PADs make it an ideal target for inhibition.

## Supplementary Material

Web supplement
