# Supplementary material for: Crystal structure of Porphyromonas gingivalis peptidylarginine deiminase: implications for autoimmunity in rheumatoid arthritis
Source: Ann Rheum Dis. 2015 Jul 24;75(6):1255–61. doi: 10.1136/annrheumdis-2015-207656 (PMC4893104; doi:10.1136/annrheumdis-2015-207656)
Supplement: Web supplement [file annrheumdis-2015-207656-s1.pdf]

SUPPLEMENTARY INFORMATION

Crystal structure of *Porphyromonas gingivalis* peptidylarginine deiminase: Implications for autoimmunity in rheumatoid arthritis

Anna B. Montgomery[a], Jolanta Kopec[b], Leela Shrestha[b], Marie-Laetitia Thezenas[c], Nicola A. Burgess-Brown[b], Roman Fischer[c], Wyatt W. Yue[b,*], Patrick J. Venables[a,*]

[a]Kennedy Institute of Rheumatology, Nuffield Department of Orthopaedics, Rheumatology and Musculoskeletal Sciences, University of Oxford, OX3 7FY
[b]Structural Genomics Consortium, Nuffield Department of Clinical Medicine, University of Oxford, OX3 7DQ
[c]Target Discovery Institute, Nuffield Department of Medicine Research Building, University of Oxford, OX3 7FZ

*To whom correspondence should be addressed:
W.W.Y. (wyatt.yue@sgc.ox.ac.uk)
P.J. Venables (patrick.venables@kennedy.ox.ac.uk)

Keywords: Rheumatoid arthritis, ACPA, Periodontitis, Citrullination, PAD

Abbreviations

PAD, peptidylarginine deiminase; PPAD, *Porphyromonas gingivalis* PAD; PD, periodontitis; ACPA, anti-citrullinated protein/peptide antibodies; RA, rheumatoid arthritis; aa, amino acid; GME, guanidino modifying enzyme; RMSD, root mean square deviation; WT, wild-type; LC-MS, liquid chromatography-mass spectrometry

Content

Supplementary Materials and Methods

Supplementary Table S1

- Figure S1 (attached TIFF file)
- Figure S2 (attached TIFF file)

Supplementary Figure Legends

Supplementary References

Table S1: Crystallography refinement statistics.

	**tPPAD**^{WT}	**tPPAD**^{C351A}
PDB code	5ak7	5ak8
**Data collection and processing**		
Beamline	Diamond I03	Diamond I03
Wavelength (Å)	0.97625	0.97625
Unit cell parameters (Å)	101.860 84.660 55.660	101.79 84.09 55.460
(°)	90.0 92.5 90.0	90.0 92.64 90.0
Space group	C2	C2
Resolution range (Å)	65.08-1.46 (1.50-1.46)	64.8-1.48 (1.52-1.48)
Observed/Unique reflections	272195/79913 (18093/5724)	253844/73831 (18948/5330)
Rsym(%)	4.1 (49.7)	5.5 (53.5)
I/sig(I)	14.3 (2.2)	10.4 (2.1)
Completeness (%)	97.9 (95.0)	95.3 (93.9)
Multiplicity	3.4 (3.2)	3.4 (3.6)
**Refinement**		
Rcryst (%)	12.71	12.72
Rfree (%)	15.32	16.69
Wilson *B* factor (Å^2)	13.92	15.199
Average total *B* factor for protein(Å^2)	18.13	19.03
Average ligand *B* factor (Å^2)	27.90	24.68
R.m.s.d. bond length (Å^2)	0.0061	0.0127
R.m.s.d. bond angle (°)	1.2697	1.5588
**Molprobity analysis**		
Ramachadran favoured (%)	97.64	97.64
Ramachandran outliers (%)	0	0

Data for highest resolution shell are shown in parenthesis.

MATERIALS AND METHODS

Sodium dodecyl sulphate-polyacrimide gel electrophoresis (SDS-PAGE)

Samples were prepared for electrophoresis with addition of Novex® NuPAGE® 4x LDS sample buffer (LifeTechnologies) and 0.1 M Dithiothreitol (DTT). Proteins were denatured by heating at 95°C for 5 minutes before resolution on 12-14% Novex® NuPAGE® Bis-Tris gels (LifeTechnologies) in 1x MOPS running buffer (LifeTechnologies) at 125 V for 45 minutes. For electrophoresis of smaller peptides, samples were prepared with 2x Tricine sample buffer and 0.1 M DTT, heating as before and resolution on Novex® 10-20% Tricine Gels in Tricine SDS running buffer (all LifeTechnologies) at 125 V for 90 minutes. Following electrophoresis, proteins were visualized by gel staining with Coomassie, or transferred to nitrocellulose membranes at 72 V for 3 hours at 4°C for immunoblotting.

Colourimetric detection of citrulline.

The colorimetric assay for citrullination activity was used as previously described [1]. Briefly, specified concentrations of enzyme were incubated in 96 well Co-star plates with 20mM concentration of benzoyl arginine ethyl-ester (BAEE) (Sigma), 5mM concentration of peptides Arg-Gly-Glu, Met-Arg-Phe, Gly-Arg, FibA-R (CESSSHHPGIAEFPS-R) and FibA-R-XX (CESSSHHPGIAEFPS-R-GK) (Pepceuticals) in PPAD buffer (50mM CHES, pH 9.5, 20mM DTT) or PAD buffer (200mM Tris-HCl, pH 7.6, 20mM $CaCl_2$, 20mM DTT) for 1 hour at 37 °C. Substrate only wells were used for blank measurements and a standard curve of 0-500μM L-citrulline included for calculations. Citrulline produced was measured by addition of a 1:3 ratio of developing reagent A (80mM 2,3-

Butanedione oxime (DAMO), 2mM Thiosemicarbazide (TSC)) to reagent B (3M 85% H_3PO_4), 6M 96-98% H_2SO_4, 2 mM $NH_4Fe(SO_4)_2 \cdot 12H_2O$ (Ammonium ferric sulphate Dodecahydrate)) and incubation at 95°C for 15 minutes. The plate was cooled briefly on ice before absorbance was measured at 540nm. Citrulline produced was calculated using absorbance reading of the L-citrulline standard curve in Microsoft Office Excel. Statistics were calculated using GraphPad Prism 6.0.

Cleavage of fibrinogen and human α-enolase by arginine gingipain B.

Recombinant human proteins fibrinogen and alpha-enolase (Sigma Aldrich Cat no: F3879, MyBioSource Cat no: MBS203689) were prepared to a working concentration of 1 mg/ml as per the manufacturer's instructions. Recombinant *P. gingivalis* arginine gingipain B (RgpB) was kindly donated by Dr Ky-Anh Nguyen following purification as described [2]. For optimum Rgp activity, a 1:10 ratio of Rgp:protein was incubated at 37°C for 24 hours before proteins were separated by SDS-PAGE for Coomassie staining and immunoblotting. Where citrullination was to be investigated, the same protocol was observed with the addition of an equal quantity of PPAD to RgpB, or with the replacement of Rgp by PPAD.

SUPPLEMENTARY FIGURES

Figure S1: Secondary structure assignment for PPAD. Vector-encoded amino acids are underlined in the sequence. Residues as part of the tPPAD construct but not observed in electron density map, and hence not modelled in the structure, are in lower case.

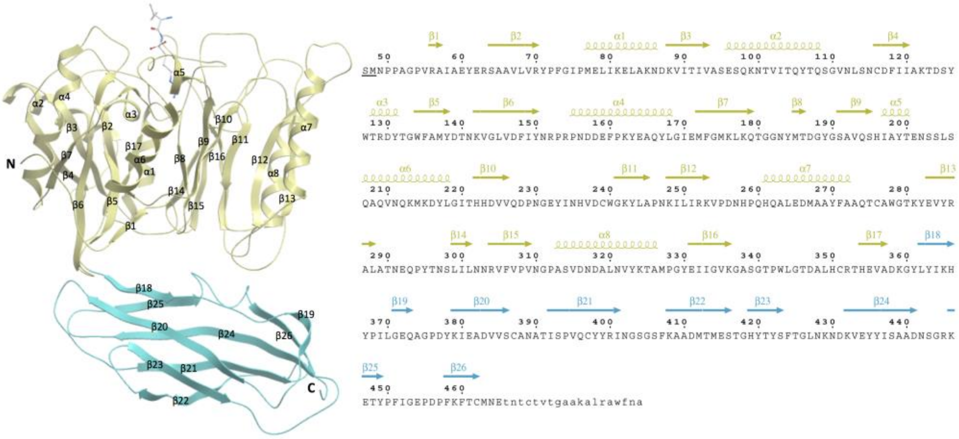

Figure S2: Proposed enzymatic mechanism of PPAD in the conversion of C-terminal arginine to citrulline. Role of PPAD residues is shown in blue. Enzyme states captured by the crystal structures of $tPPAD^{C351A}$ and $tPPAD^{WT}$ are indicated

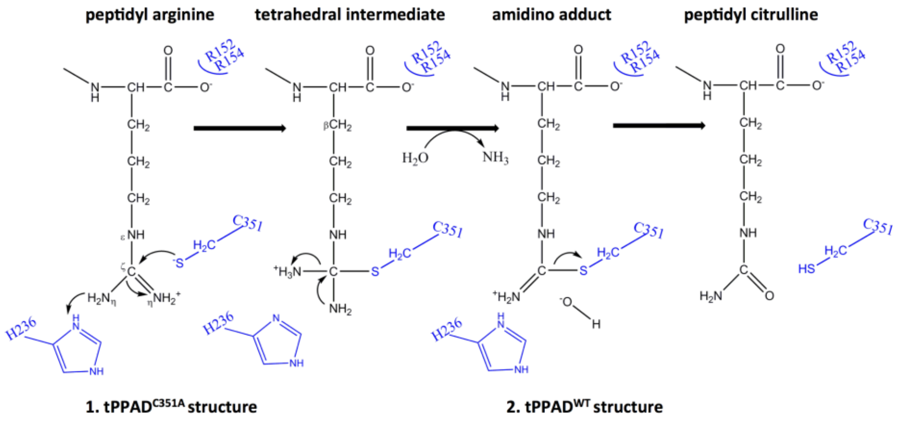

SUPPLEMENTARY REFERENCES

1. Rodriguez SB, Stitt BL, Ash DE. Expression of peptidylarginine deiminase from Porphyromonas gingivalis in Escherichia coli: enzyme purification and characterization. Arch Biochem Biophys 2009;**488**(1):14-22 doi: 10.1016/j.abb.2009.06.010 [published Online First: Aug 1].
2. Veillard F, Potempa B, Guo Y, et al. Purification and characterisation of recombinant His-tagged RgpB gingipain from Porphymonas gingivalis. Biol Chem 2015 doi: 10.1515/hsz-2014-0304 [published Online First: Feb 4].
